# Obtaining Patient-Reported Outcomes Electronically With “OncoFunction” in Head and Neck Cancer Patients During Aftercare

**DOI:** 10.3389/fonc.2020.549915

**Published:** 2020-11-25

**Authors:** Veit Zebralla, Juliane Müller, Theresa Wald, Andreas Boehm, Gunnar Wichmann, Thomas Berger, Klemens Birnbaum, Katharina Heuermann, Steffen Oeltze-Jafra, Thomas Neumuth, Susanne Singer, Matthias Büttner, Andreas Dietz, Susanne Wiegand

**Affiliations:** ^1^Department of Otorhinolaryngology, Head and Neck Surgery, University Medical Centre, Leipzig, Germany; ^2^Innovation Center Computer Assisted Surgery, University of Leipzig, Leipzig, Germany; ^3^Department of Otorhinolaryngology, Clinic St. Georg Leipzig, Leipzig, Germany; ^4^Fraunhofer-Institute for Photonic Microsystems, Dresden, Germany; ^5^Institute of Medical Biostatistics, Epidemiology and Informatics (IMBEI), University Medical Center Mainz, Mainz, Germany

**Keywords:** patient-reported outcome, questionnaire, head and neck cancer, follow-up, Patient-Reported Outcome Measure, “OncoFunction”

## Abstract

The disease and treatment of patients with head and neck cancer can lead to multiple late and long-term sequelae. Especially pain, psychosocial problems, and voice issues can have a high impact on patients’ health-related quality of life. The aim was to show the feasibility of implementing an electronic Patient-Reported Outcome Measure (PROM) in patients with head and neck cancer (HNC). Driven by our department’s intention to assess Patient-Reported Outcomes (PRO) based on the International Classification of Functioning during tumor aftercare, the program “OncoFunction” has been implemented and continuously refined in everyday practice. The new version of “OncoFunction” was evaluated by 20 head and neck surgeons and radiation oncologists in an interview. From 7/2013 until 7/2017, 846 patients completed the PROM during 2,833 of 3,610 total visits (78.5%). The latest software version implemented newly developed add-ins and increased the already high approval ratings in the evaluation as the number of errors and the time required decreased (6 vs. 0 errors, 1.35 vs. 0.95 min; *p*<0.01). Notably, patients had different requests using PRO in homecare use. An additional examination shows that only 59% of HNC patients use the world wide web. Using OncoFunction for online-recording and interpretation of PROM improved data acquisition in daily HNC patients’ follow-up. An accessory timeline grants access to former consultations and their visualization supported and simplified structured examinations. This provides an easy-to-use representation of the patient’s functional outcome supporting comprehensive aftercare, considering all aspects of the patient’s life.

## Introduction

Advanced head and neck cancer (HNC) mostly requires an aggressive multimodal treatment approach based on tumor resection and/or radio-chemotherapy. Besides causing early toxicities, these may lead to multiple late-term impairments and often seriously affect quality of life (QoL). In recent years, the survival rate of a subgroup of HNC patients has improved due to the higher vulnerability of human papillomavirus (HPV)-associated disease to cisplatin-based radio-chemotherapy and the development of new surgical, radio-therapeutic and molecular therapy strategies. Therefore, therapy-related impairments are becoming increasingly relevant. Patients undergoing treatment of HNC are usually included in standardized (NCCN-guideline conform) follow-up programs, recommended for five years (1, 2). The purposes of post-therapeutic aftercare are early identification of recurrence, early detection of secondary primary cancer in the head and neck area, description of functional impairments, and management of complications (3). However, there is no high-level evidence for particular follow-up practices. Follow-up visits regularly focus on the detection of recurrence but usually are less attentive to the perception and documentation of functional impairments and QoL. Though, especially patients suffering from HNC experience a wide variety of disease- and treatment-related functional impairments, such as swallowing problems, voice problems, depending on the use of tracheostomy tube and gastrostomy tube, as well as psychosocial problems, pain, loss of employment, and aesthetic impairments [3]. Therefore, a patient-centered approach to comprehensively assess the impact of treatment and care on function and QoL is necessary and should provide actionable information to the treating physician in near time, at best immediately.

For the recording of patient-reported outcome (PRO) utilizing various instruments in daily clinical practice has been published (4–8), and also the electronic application (ePRO) using mobile devices has been described for other tumor entities (9). A PRO is defined by the US Food and Drug Administration (FDA) as “any report of the status of a patient`s health condition that comes directly from the patient, without interpretation of the patient`s response by a clinician” (10). By using PRO Measurements (PROM), the patients’ perspective about functional status, health status, symptoms, QoL, psychological and social well-being can be assessed unfiltered. Therefore, PRO instruments are one of the most efficient tools for quantifying patients’ experience. For solid metastatic tumors (excluding head and neck squamous cell carcinoma, HNSCC), an improvement in overall survival (OS) compared to usual care was demonstrated using PRO (11).

Knowing PRO can optimize communication, symptom management, and increase satisfaction with applied treatment (12) and considers (e.g., psychiatric or psychooncologic) comorbidities. Within the scope of clinical trials, the use of PROM also is increasingly requested. Only the use of standardized function-based assessment tools prevents the risk caused by using different measurement instruments (for example, Hospitality Anxiety and Depression Scale (HADS) vs. PHQ-9 and GAD) and the potential interferences of physical with psychosocial factors as previously described by Singer et al. (13, 14).

Despite their positive aspects, the use of PROM and electronic PROM in clinical routine is at the moment unusual in the context of HNC aftercare, probably due to missing evidence regarding benefit for the patient and cost-effectiveness in scientific literature. Believing in advantages of standardized PROM included in regular visits over unstructured interviews during follow-up visits, our university’s ENT-department contributed to the development of the software “OncoFunction” that since 2013 is used to evaluate PRO in HNC patients’ tumor aftercare (15). This report outlines 1) the implementation of the system, 2) the feasibility of its use in HNC patients, 3) newly developed and implemented add-ins, and 4) presents data of an evaluation trial with clinical experts showing the benefit of these developments. Additionally, we would like to create a view in the future and evaluate if home-based PROM solutions for follow-up assessments in HNC patients are likely feasible.

## Material and Methods

### The Instrument

The PROMs, collected with the software “OncoFunction”, are based on the International Classification of Functioning (ICF) of the World Health Organisation (WHO) (16). Parts of the ICF-core set were defined and evaluated by Tschiesner et al. and Stier-Jarmer et al. (17, 18), and a clinical practice guideline for tumor aftercare in HNC patients was published and recommended by the German Cancer Society in 2013 (19). This guideline includes the proposed questionnaires. The selection of the questions based on medical experts consents and mostly validated screening instruments were implemented in the PRO. Patients were not involved in the development of the PROM. The questionnaire in “OncoFunction” focuses on the central problem areas for HNC patients, which are pain, swallowing, voice and breathing, psychosocial terms, and other problems (20). To detect problems in pain the numeric analogue scale is used, to collect data about swallowing problems the EAT-10 is implemented. Screening for depression and anxiety is realized with the PHQ-9 and the GAD-2 questionnaire. Voice, fatigue, and global quality of life are captured with the corresponding items from the EORTC QLQ C30. Five additional questions were included to record: 1) presence of tracheal tube, 2) laryngectomy and presence of an artificial voice, 3) smoking, 4) alcohol consumption, and 5) employment status. The second part of the instrument is a physician’s questionnaire and checklist, where the clinical findings and therapeutic consequences are documented. Documentation includes the results of the clinical examination, the Common Terminology Criteria for Adverse Events (CTCAE v. 4), and the Eastern Cooperative Oncology Group Performance Status Scale (ECOG-PSS). The development of the screening tool and the standardized functional evaluation were introduced by Kisser et al. in 2016 (20).

#### The Electronic System and the Clinical Execution

The software “OncoFunction” was implemented and provided by the company IMPULS Technologiemanagement UG, Leipzig, and is used in a local internal network at our ENT-department. In this network, eight tablet computers (Samsung Galaxy 10n.1) and one server were registered and connected by a wireless LAN router. The questionnaires and the visualization of the results are programmed in a web-browser-based solution (Microsoft Internet Explorer 10). The results of the patient’s questionnaires are immediately recorded and interpreted, and the calculated scores are visible for the clinician right after completion of “OncoFunction”. All data is saved on a server for later usage.

**Figure 1:**
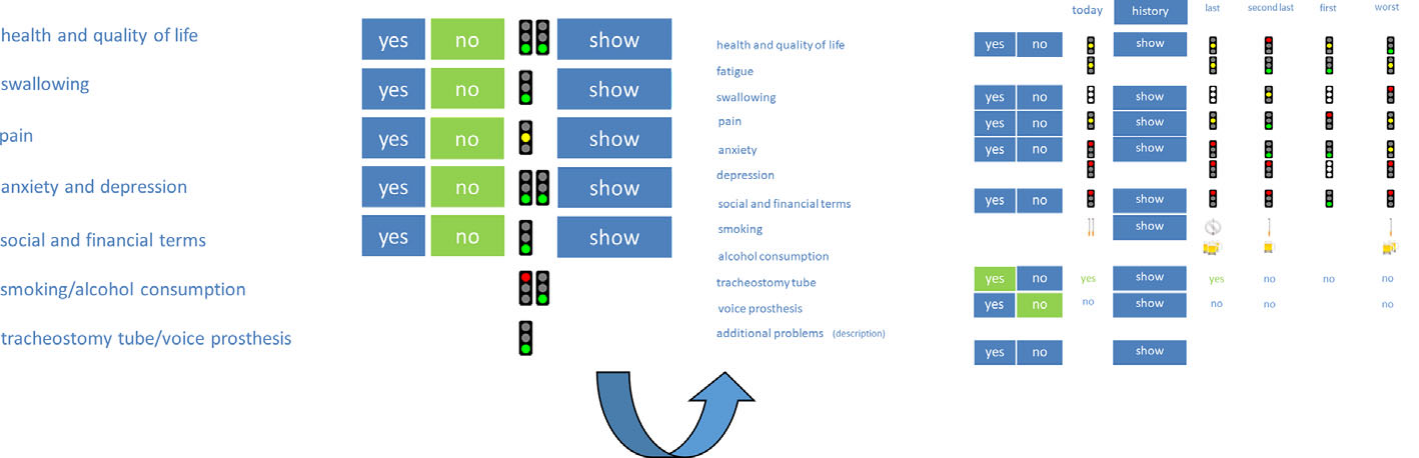
Visualization of the original questionnaire and examinations with traffic lights. Red color symbolizes a lousy condition, yellow color, a moderate condition, and green color, a good condition of the patient.

“OncoFunction” is used right before every follow-up visit for every patient suffering from HNC. The follow-up schedule is based on the recommendations for head and neck cancer follow-up in the NCCN guidelines. In the first year, visits were conducted every 1-3 months, in the second year every 2-6 months and in year 3-5 every 4-8 months. Patients with cancer history longer than 5 years have annual follow-up visits. No tumor entity, stage or head and neck tumor site is excluded in our aftercare; thus, all patients were encouraged to complete the PROM regularly. This means that both curatively and palliatively treated patients were invited to fill out the questionnaire. However, participation is voluntary. A nurse documents the patient ID and additional parameters like age (to verify the patient’s ID), height and weight (to calculate the body mass index), and the patient’s informed consent to (further) participate in the study. After registration and obtaining informed consent according to the studies protocol as approved by the ethics committee of the Medical Faculty (vote no. 201-10-12072010 and no. 202-10-12072010), the nurse delivers the tablet computer to the patient. All subjects signed informed consent in accordance with the Declaration of Helsinki. If the patient asks for support to fill in the required information, the nurse assists in handling the tablet PC to record the answers. The collected data is sent online to a single server designed to process and store the data in a secure environment. The results are graphically visualized for the physician. Important patient-specific problem areas can easily be detected, and an intervention can be initiated. In the first and original version, a traffic light presentation was used to visualize the results of the patient questionnaire (**Figure 1**). A red color indicates the need for attention, a yellow color states a moderate condition, and a green light shows a probably good situation. The cut-off values for the light’s visualization were defined in a consensus conference and published by Kisser et al. (20). In the last software update, the traffic light presentation was changed to a face-like icon presentation. After the clinical assessment, the physician documents the clinical findings and fills out a checklist for the examination.

Initially, the use of our PROM started 8 weeks after therapy in the setting of tumor aftercare. Thus, one could not differentiate between disease-specific and therapy-induced impairments. Meanwhile, patients answer the PROM during the evaluation of diagnosis on the day before panendoscopy to document disease-induced impairments, too.

### The First Innovation of “OncoFunction”

After the implementation of the system, it became apparent that some parts need to be changed to create better usability of the system. In the first version, there were no timelines or results of older examinations and questionnaires visible. Thus, the visualization of previous examinations with traffic lights was added to evaluate the direction of the functional development in clinical practice. The possibility to compare current functional impairment(s) with earlier findings helps to track the functional development faster and highlights increasing impairment. In the physician’s part of the software, the documentation was simplified. Instead of a free field answer of chosen interventions, a drop-down menu with proposals of interventions has been integrated. This version was enrolled in July 2015.

### Implementation of New Functions and Optimized Visualization of “OncoFunction”

**Figure 2:**
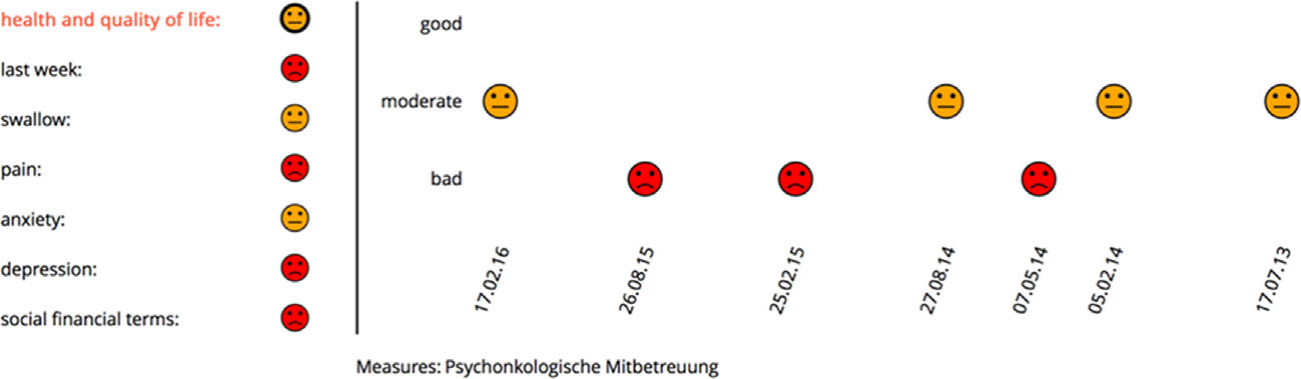
New visualization realized with face-like icons. A red icon indicates problems and a need for attention, a yellow icon, a moderate condition of the patient, and a green icon in a good situation. In this case, psycho-oncological support was started concerning many problematical findings in the problem field health and quality of life.

**Figure 3:**
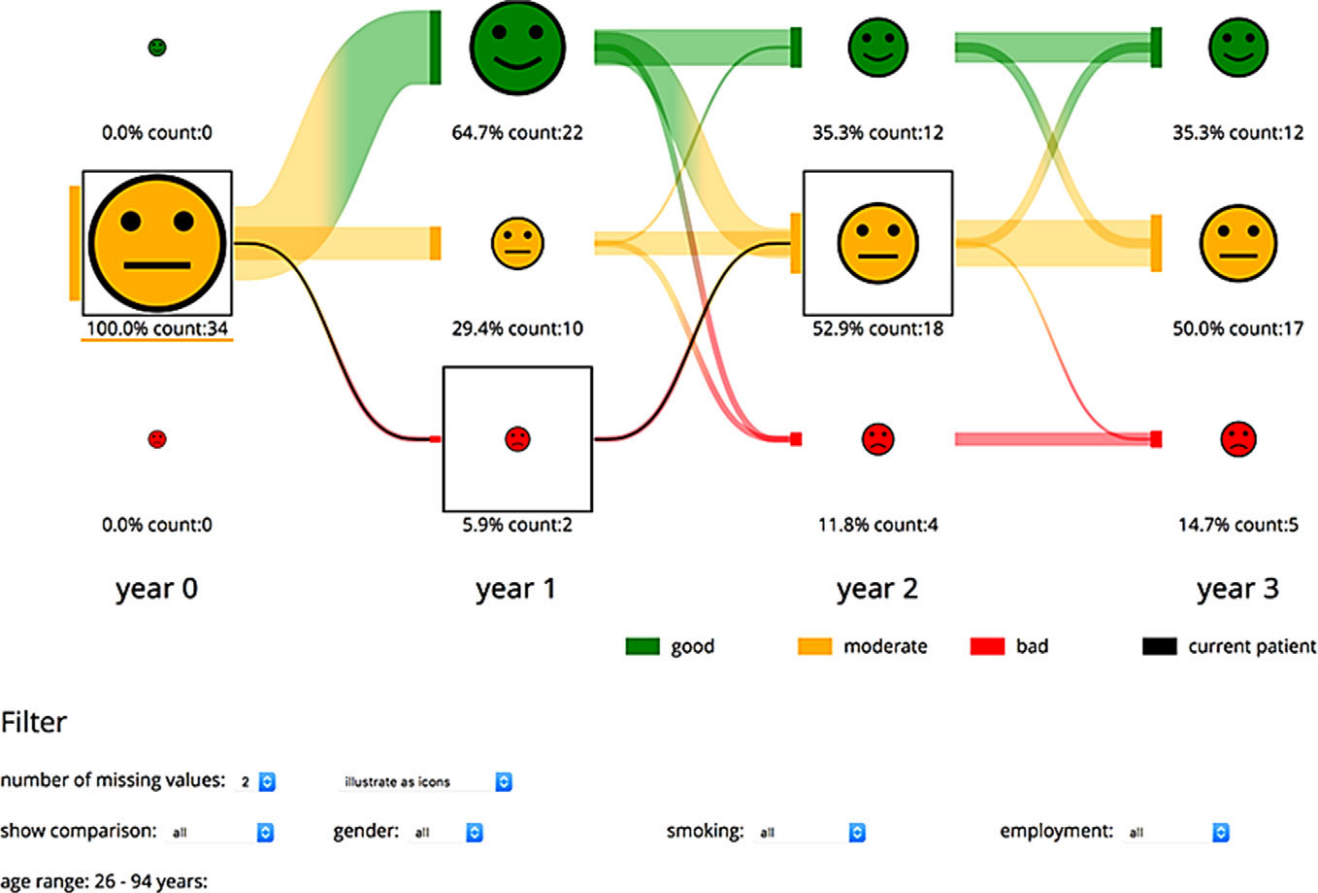
Visualization of the progress of one patient compared to a selected patient group. The physician can choose the currently visualized function domain and patient group. The number of patients is represented through the icon size and additionally given as text. The development of the patients over the years is illustrated through lines between each state. The development of the current patient is given through the black lines and boxes.

The latest software version focused on increasing clarity and implementing new functions in the program. To reduce the visible items on the first slide, the traffic light encoding was changed into a presentation based on face-like icons (21). Here, the initial view is split into two views: the left depicts the last state of patients’ functional aspects, whereas the right part displays the development of the patient in the currently chosen functional aspects on display. Through tooltip methods on the icons on the left side, the visualized development in the functional domain can be changed. Thus, the screen is clarified, and the interpretation of data visualized is more intuitive (**Figure 2**). The new version allows for showing a patient’s progress either per consultation or per year. Additionally, a patient’s course of symptoms up to 3 years after therapy in comparison to other participating patients can be visualized. Through selection methods, the group of comparison can be filtered (**Figure 3**). With these new specifications, co-findings and individual risk factors leading to specific functional changes after treatment of head and neck tumors can be identified and may sensitize for finding ways to prevent functional impairment.

### Evaluation Study

We executed an evaluation study with 20 physicians and medical researchers to evaluate the latest program update and developments. In this survey, the test persons had to negotiate some tasks in the old (initial) and new (latest) version of “OncoFunction”. These tasks were:

1. Mark the current value in the functional aspect of “health and quality of life”.
2. Mark the patient’s development from the last to the current value in the functional aspect “pain”.
3. Mark the answer to the penultimate question “dysphagia weight loss” in the functional aspect “swallowing”.
4. Mark the suspicious answers.

The time required and the number of errors performed during each task was documented. Afterward, questions related to clinical and technical evaluation were answered by the test person. These questions were:

1. Do you think the collection of functional aspects (swallowing, QoL, etc.) is useful?
2. In case of a red marked item, would you ask for current problems?
3. What is the best way to collect the most information about the patient?
4. Are more problem areas addressed with the usage of the PRO compared to regular aftercare consultation?
5. Is the new visualization helping to speed up investigation of problem areas?
6. Do you think that the visualization of functional development over time is useful for your clinical work?
7. Is the usage of PROMs justifying the additional required time to investigate all problem areas?

The trial was realized in a one-to-one single-blind setup, where the test person not familiar with the use of “OncoFunction” did not know which OncoFunction version was the latest one tested the software version in randomly appearing order.

### Home Application of Online ePROMS in HNC Patients

To evaluate the acceptance and potential usage of home-based, online available ePROMS, questionnaires were submitted to tumor aftercare patients consisting of questions concerning their internet use and the qualities and limits of answering an online questionnaire. All patients were invited to answer 12 questions to their online behavior. These questions were in detail:

1. Sex and age?
2. What is your highest degree in education?
3. Do you own an internet-based device?
4. If yes, what kind of device?
5. Do you use the internet?
6. Could you imagine communicating *via* APP with your physician/clinic?
7. As a patient treated for cancer or chronic disease in aftercare: How often would you like to answer questionnaires?
8. How much time you spend would be comfortable?
9. How many questions would you agree to answer regularly?
10. Would you be able to collect personal data (e.g., state of health, height, weight, heart rate, blood pressure, etc.), if necessary, supported by the APP?
11. Do you think that using an APP would improve patient care?
12. Would you use an APP to transfer your health data to your hospital?

### Statistical Analysis

We report frequencies (n) and column percentages (%) for categorical characteristics and means (M) and standard deviations (SD) for continuous characteristics. Chi-square tests were used to explore associations between the respective two groups and the categorical characteristic. To examine associations between continuous variables, we used t-tests. For all statistical tests, the result is considered as statistically significant with a 2-sided type-I-error-probability α<0.05.

## Results

### Implementation of the System

**Figure 4:**
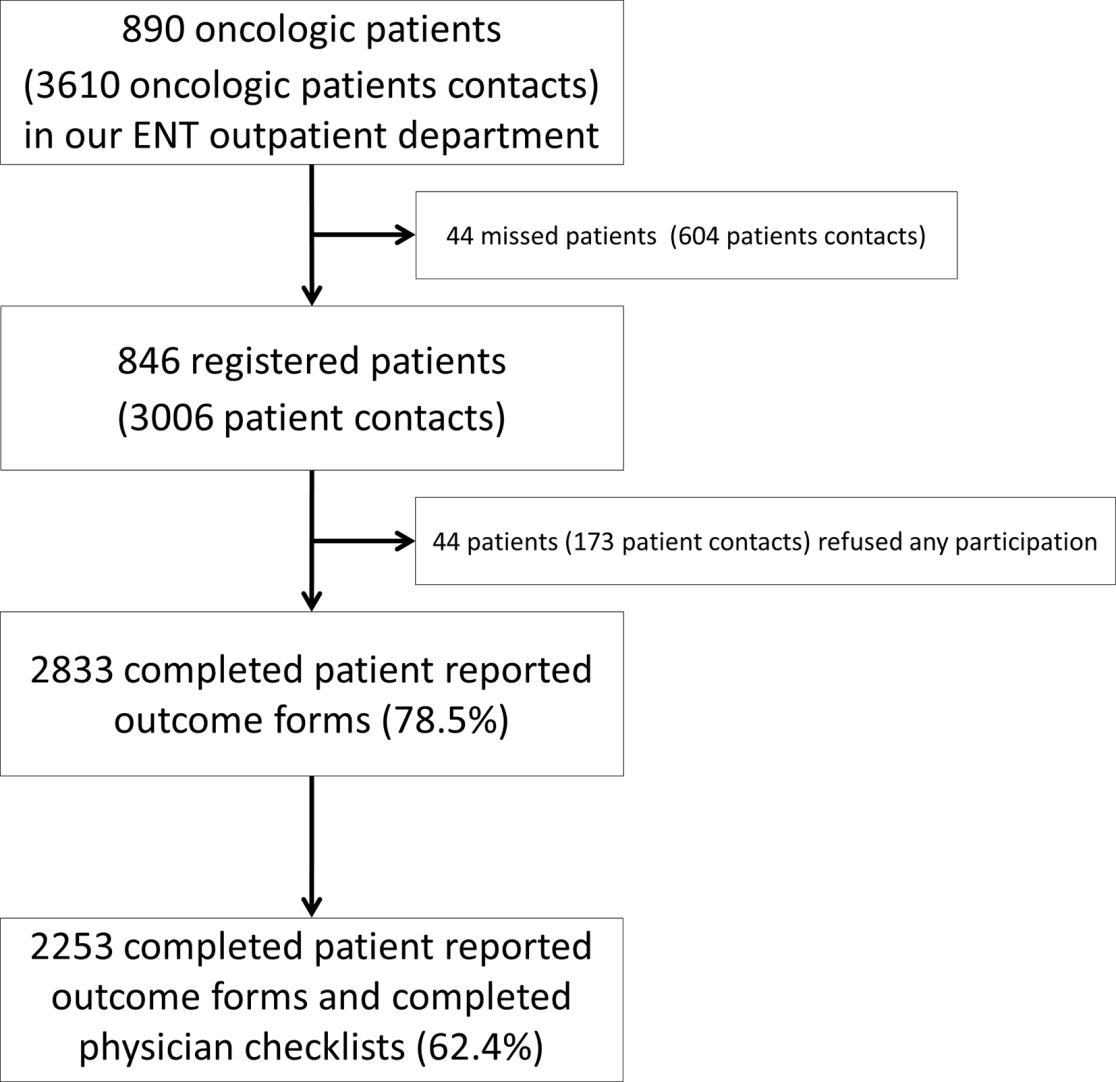
Flowchart of patient recruitment in our clinic from 7/2013 until 7/2017.

From 07/2013 until 7/2017, 3006 registered patient contacts had been documented in the database initiated by 846 patients (**Figure 4**).

Among the 846 patients included, 657 were males and 189 females. Patients’ characteristics are shown in **Table 1**. In the same period, a total number of 890 different oncological patients with 3,610 physician-to-patient contacts had been registered in our ENT-Department. All oncological tumor sites and stages were included in this trial. In 802 patients, we collected 2,833 complete questionnaires. 173 contacts led to no recording of data as some patients refused the participation (3.6%) or were unable to answer the questionnaire at some time (2.2%). “OncoFunction” was used in 78.5% of all cases. The rate of completed physician checklists was lower than the rate of the answered patient questionnaires. A total number of 2,253 checklists (73%) were complete (**Table 2**).

**Table 1:** Patient characteristics.

Total n=846		Male n=657 (77.7%)	Female n=189 (22.3%)
Age in years Tumor localization		63 ± 11	62 ± 11
Larynx (C32)	Total n=166 (19.6%)	**146**	**20**
	pTis	1	0
	UICC I	39	1
	UICC II	25	2
	UICC III	26	11
	UICC IV	55	6
Hypopharynx (C13)	Total n=89 (10.5%)	**85**	**4**
	UICC I	2	1
	UICC II	5	0
	UICC III	12	0
	UICC IV	65	3
	Unclear	1	
Oropharynx (C01, C05, C09, C10)	Total n=305 (36.1%)	**234**	**71**
	UICC I	21	9
	UICC II	15	4
	UICC III	34	15
	UICC IV	163	42
	Unclear	1	1
Nasopharynx (C11)	Total n=17 (2%)	**8**	**9**
	UICC I	0	1
	UICC II	0	1
	UICC III	3	3
	UICC IV	4	4
	Unclear (no UICC – State)	1	
Tongue (C02)	Total n=61 (7.2%)	**42**	**19**
	UICC I	11	7
	UICC II	4	3
	UICC III	11	2
	UICC IV	16	7
Floor of mouth (C04)	Total n=33 (3.9%)	**26**	**7**
	UICC I	4	2
	UICC II	4	2
	UICC III	3	1
	UICC IV	15	2
Cervical CUP (C80)	Total n=36 (4.3%)	**29**	**7**
	UICC III	1	4
	UICC IV	28	3
Other (C03, C07, C08, C15, C30, C31, C41, C43, C44, C73 C83)	Total n=139 (16.4%)	**87**	**52**

**Table 2:** The absolute number of answered patient’s questionnaires and clinician’s checklists (total contacts n=3,006).

	Patient answered questionnaire n=2,833 (94.2%)	Patient refused questionnaire n=108 (3.6%)	Patients reduced general condition n=65 (2.2%)
Completed clinician’s checklist n=2,253	2193 (73%)	45 (1.5%)	15 (0.5%)
Missing clinician’s checklist n=753	640 (21.3%)	63 (2.1%)	50 (1.6%)

The mean number of recorded questionnaires per patient was 3.6 (range 1–13) during the follow-up time (**Table 3**).

**Table 3:** Number of answered questionnaires by the patients during their follow-up visits.

Number of answered questionnaires using “OncoFunction”	1	2	3	4	5	6	7	8	9	10	11	12	13
Number of patients	235	147	108	84	74	70	53	43	19	6	5	0	2

The duration for answering the patient’s questionnaire was recorded from 7/2013 to 7/2014. In this time, we registered 841 physician-patient contacts. We evaluated the mean time needed to fill out the questionnaire per month and calculated the mean time for answering and the standard deviation (SD). The shortest needed time was registered in 6/2014 with 8:12 min (SD 10:00 min); the longest time was 10:49 min (SD 10:08 min) in 7/2013. In summary, the times were mostly constant over the period covered.

### Evaluation of the Latest Software Version

Eleven female and 9 male medical experts took part in the evaluation trial. Of these, 14 were physicians, 5 clinical researchers, and one an oncological nurse. All participants were working in the Departments of either Otorhinolaryngology or Radiooncology. The comparison of the old and the new visualization shows that the tasks could be realized significantly faster, and fewer errors were produced using the new version (**Table 4**). Asking for the favored software version on a scale from 1 – “prefer the old version” to 6 – “prefer the new software version” results in a 4.8 ± 0.8. In this context, we evaluated the use of PROM in general within the participating colleagues. The answers were given on a numeric scale from 1 – 6. The rating for using PROM, in general, was very high, and the positive aspects of PROM were evident for most medical experts in our validation study. The detailed results are reported in **Table 5**.

**Table 4:** Comparison of old and new visualization.

n=20	Old software version	Latest software version	Significance
Time needed for tasks	1.38 ± 0.5 min	0.95 ± 0.3 min	p=0.0038*
Produced errors	6	0	p=0.0041**

*t-test; **Chi²-test.

**Table 5:** Additional questions to the medical experts concerning PROM in general with mean ± SD.

Question	Mean ± SD
Do you think the collection of functional aspects (swallowing, quality of life) is useful? (1 not useful- 6 very useful)	5.4 ± 0.9
In case of a red marked item, would you ask for current problems? (1 would not ask for it – 6 would totally ask for it)	5.7 ± 0.5
What is the best way to collect the most information about the patient? (1 classical anamnesis – 6 Patient-Reported Outcome)	3.9 ± 1.3
Are more problem areas addressed with the usage of the PRO? (1 never – 6 always)	4.9 ± 0.8
Is the new visualization helping in the fast investigation of problem areas? (1 just real slow – 6 very fast)	5.3 ± 0.8
Do you think that the visualization of functional development over time is useful for your clinical work? (1 not useful – 6 very useful)	5.1 ± 1.1
Is the usage of PROs justifying the additional required time to investigate all problem areas? (1 no – 6 yes)	4.5 ± 0.9

We also asked for additional time, which is acceptable using a system like “OncoFunction”. 12 participants answered that an additional time per patient of 1–2 min is acceptable, 4 answered 3–4 min, and another 4 that more than 5 min is acceptable.

### Home Application of Online ePROMS in HNC Patients

**Figure 5:**
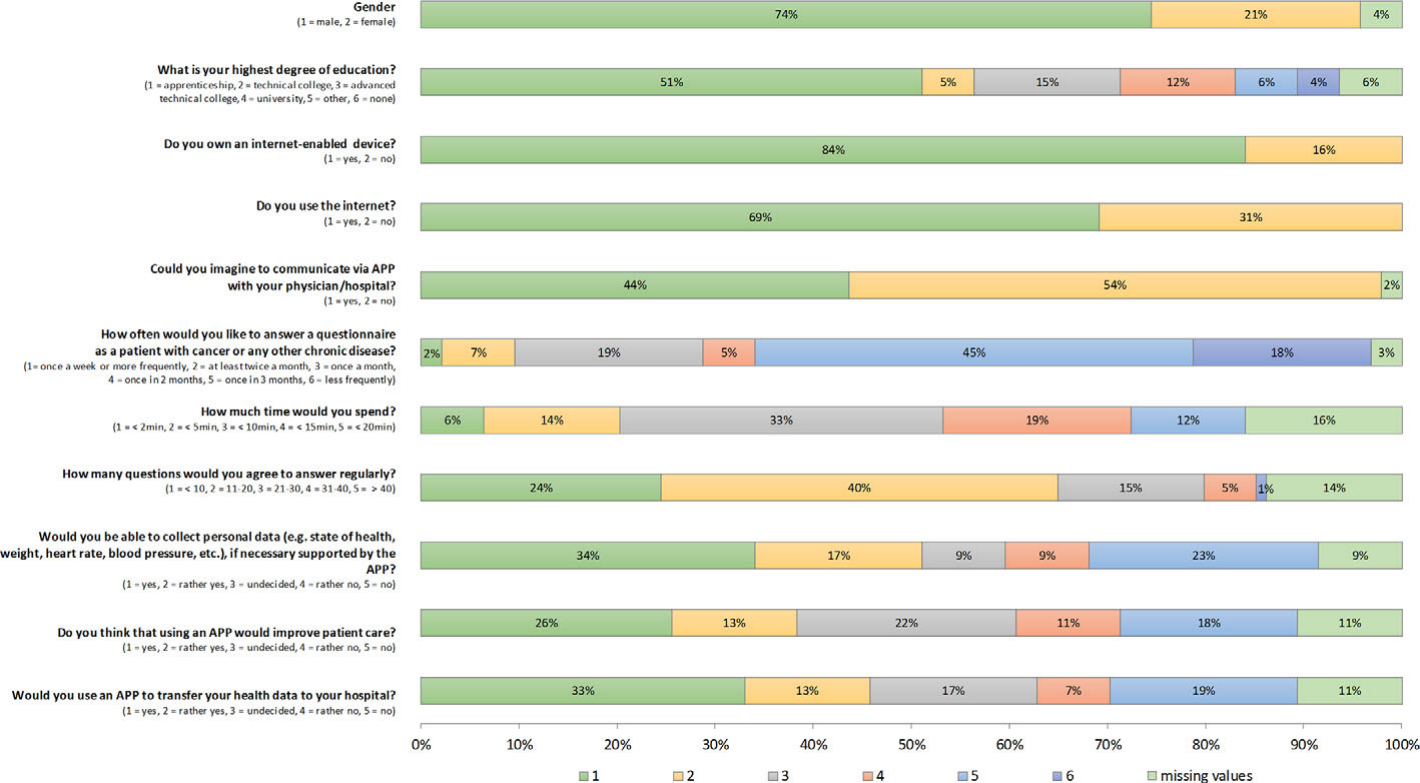
Evaluation of the patient’s view on an online, home-based electronic patient-reported outcome measure (ePROM) of 94 patients with head and neck cancer. Frequency of answers according to the questionnaire.

94 questionnaires were returned. 16% of HNC patients have no access to the internet; 31% do not use the internet. There are no significant differences in possession of an internet-enabled device, internet use, or willingness to communicate *via* APP according to age and gender (all *p >*0.115). However, patients of age < 65 years assume using an APP could lead to an improved patient care (*p*=0.013; yes/rather yes 54.5 vs. 32.4%, undecided 20.5 vs. 25.9%, no/rather no 25 vs. 29.6%). Younger patients would also agree more likely to transfer their health data *via* the APP to their treating hospital (*p*=0.024). The results are shown in **Figure 5**.

## Discussion

In the first four years using a PROM with the software “OncoFunction” in our clinic, we reached 78.5% of all physician-patient contacts. Of them, 73% also had a complete physicians’ checklist. Using the edited surface reduced the time needed to extract relevant information and error frequency significantly. In conclusion, the implementation of the tool was successful.

However, the successful implementation of an ePROM in the clinical practice of head and neck tumor aftercare is challenging and resource-consuming despite its positive aspects. The PROM is a reliable measurement instrument for assessing quality of life, a symptom and symptom strength assessment tool, and overall a health status reporting system that significantly improves the patient–physician communication (22), optimizes supportive care (23), and therefore can lead to positive aspects on the shared decision-making process (12). The visualization of problems and the possibility to see the need for intervention in a well-structured intuitively interpretable form allows for getting the required information from the patient much quicker. Problems can be identified faster, more precisely, and decisions are made more patient-centered. After usage of the PROM in clinical practice, most patients were willing to use this instrument in the next tumor aftercare consultation again. In a 2016 published feasibility study of “OncoFunction”, 202 patients were asked about issues concerning the usability and the patient’s view on usefulness of the tool. Patients confirmed intuitively use and meaningfulness of the tool. Moreover, participating patients described a better response to their problems and a high willingness for recurrent use (15). Different trials have examined the impact of the use of PROs on patient-physician communication. In line with our findings, specific issues related to PROs, such as symptoms and psychosocial issues, were more likely to be discussed (24).

However, the implementation of a new system has to overcome barriers, too. It was necessary to include and train all staff members to run the project successfully. In several information sessions, the goals and the way to get positive results (mainly to reach as many participants as possible and have them completing the questionnaires) were communicated, and concerns were discussed. The results and problems were evaluated to optimize the system in clinical practice use. This led to a high acceptance rate of the ePROM in our clinic. OncoFunction allowed for collecting data from 78.5% of all patients in our regular tumor aftercare. In a cross-sectional survey on cancer survivors in England, analyzing the feasibility of collecting population-based PROM, only 66% of all patients returned completed questionnaires (25). In a current publication, similar data had been shown by Duman-Lubberding et al. using the system OncoQuest which is also an example for a successful implementation of a PROM in the regular clinical aftercare (26). This OncoQuest system was implemented in 2006 in a routine clinical setting in Amsterdam (27). Their system monitors the health-related quality of life. In sum, 79 questions were asked to the participant by OncoQuest derived from the EORTC QLQ-C30 and QLQ-HN35 questionnaires and the Hospital Anxiety and Depression Scale (HADS). The physician also can see answers and of them calculated values in real-time on a screen. The most commonly reported barriers not using their OncoQuest system were lack of time, no questions regarding supportive care, and absence of symptom change (26).

The gap between participating patients and thoroughly answered questionnaires in our study results from system instability after initial implementation and the temporary collapse of the electronic system, leading to the missing data record of an entire day. The high rates of repeatedly answered questionnaires show that patients are motivated to use the ePROM in clinical routine and contribute to complete time series. Moreover, the highlighted changes led to anamnestic clarification and often focused examination of organ function and cause of reported changes by the treating physician (unpublished).

To map the status quo before treatment, the use of “OncoFunction” now starts at the time of diagnosis, which was defined as the day before panendoscopy, and facilitates the functional examination. Starting with the PROM before therapy is very important to provide a relevant baseline value: first, there is a difference in functional deprivation according to the tumor site and the tumor stage, which must be attended. Second, the acceptable functional impairment by therapy differs affected by many aspects like age, clinical condition but also by further functional deprivation. As a scenario in the future, the use of pretherapeutic assessed PROs could influence decision making for further therapy, based on pretherapeutic reported functional impairments and their relation to later on decreasing, persisting or even increasing functional impairment observed after particular treatment.

Cognitive dysfunction through severe alcohol abuse occurs more often in head and neck tumor patients than in other entities. Korsakow’s syndrome resulting from alcohol abuse is a specific problem because patients with this disease are mostly unable to give proper answers to the questions. Also the rate of illiterate and patients with suboptimal health literacy is higher in the group of head and neck tumor patients, than in other entities (28). For illiterate patients, a PROM, without potential bias (for instance, by an assisting interviewer), is challenging, and to use “OncoFunction” may not be the optimal way of tumor aftercare for these patients. If the claim is to get information from this subgroup, a higher number of nurses may be needed to assist those patients while answering the questionnaire. That is why there may be a selection bias (29). In our cohort the HPV rate in oropharyngeal carcinomas was not examined. For the implementation of aftercare systems, especially online tools, this information might be relevant due to the reported younger age of patients with HPV related oropharyngeal carcinomas.

It was suggested by a previous study that there is no significant difference between tumor site and stage in HNC patients participating or not participating in using PROMs (15). Duman-Lubberding et al. also examined the feasibility of an ePROM (“OncoKompas”) in a small study among 56 head and neck cancer survivors, and showed that there was no difference regarding patient-specific variables (TNM, age, gender, tumor location, comorbidity and quality of life (QoL)) (29). A follow-up paper described the successful implementation of OncoKompas in 2018 (30). “OncoKompas” is a web-based self-management tool that allows the patient to give information in a self-chosen frequency with the possibility to send a digital copy to the health care professional. This could simplify the contact with the health care professionals also in case of an occurring problem. The data of patient’s acceptance using “OncoKompas” are not published yet and so far, only 31% of the eligible hospitals in The Netherlands implemented the software tool for the trial.

Another trial, including HNC patients, showed that participants of PROM reported significantly better QoL and functioning and less severe symptoms (31).

So far, the results of aftercare consultations are difficult to compare since there are too many measurement instruments available, and the results are depending on the examiner and his description. The implementation of a PRO instrument and clear and unambiguous documentation can lead to better comparability of patient data (subjective findings and examination results) within a clinic. The subjective “patient’s reality” and objective findings are documented in the same standardized way. The results and their documentation are hence independent of the examiner, and every problem area of the patient is addressed. This will facilitate the physician’s efforts to improve individual patients’ health. With the usage of this PRO instrument in multiple hospitals, the comparability of findings between different clinics will be optimized and can provide essential data for ongoing research to improve outcomes.

It has to be mentioned that the acceptance of PROM in the daily routine practice is well. In the evaluation study, most physicians and nurses attested the positive value of using PROM for the detection of patient’s unmet needs and complete documentation. The simplified visualization allowing for a more intuitive interpretation of data leads to a better perception and improved selection of relevant information for decision-making. Most of the participants prefer the new visualized software version after the trial. Interestingly the additional time, which was judged to be acceptable (1–2 min) by most of the physicians and researchers, is higher than the time which is found to be required to solve the tasks (below 1 minute). It is generally observed that an intuitively interpretable visualization can lead to better acceptance of tools with higher rates of answered physician questionnaires (32). The interactive visualization of more significant cohort data and their comparison with individual data leads to an unusual scenario in the future: By selecting patients with parallel developments in organ function and impairment, the physician can estimate the current patient’s most probable future course in “*real-time”*.

The next future step for “OncoFunction” might be the development of an online home care system for head and neck cancer patients. For patients undergoing a hematopoietic stem cell transplantation, the feasibility and proper patient compliance, using an online tool, had been shown (33). The “OncoFunction” system as structured in-hospital solution combined with an supplementary homecare solution using a web-based application are our ideas for future developments. For the cohort of HNC patients, this also has to be discussed critically. The participants of our examination agreed only in less than 50% that the use of an online home-based tool can improve the aftercare. As an additional barrier for HNC patients, the incomplete infrastructure must be mentioned. 16% had no access, and 31% of the examined patients reported not to use the internet. Questionnaires should be kept as short and easy as possible (20 questions maximum, the time needed to answer less than 10 min). From the patient’s point of view, fulfilling a regular online ePROM once quarterly is adequate. For patients who are not willing or able to participate in online homecare programs, individual solutions to improve the aftercare must be developed.

## Conclusion

The use of an ePRO instrument in head and neck cancer patients is feasible, and the patients accepted the system “OncoFunction” in the clinical routine setting. However, the implementation of the “OncoFunction” system requires overcoming infrastructural problems to achieve acceptance by all stakeholders and keep them motivated to reach the goal of improved aftercare and earliest detection of issues. The optimized visualization and the implementation of new functions into “OncoFunction” contribute essentially to reach the goal of providing function-based, comparable, and more patient-centered tumor aftercare for head and neck patients.

## Data Availability Statement

The datasets presented in this article are not readily available because of patient confidentiality and participant privacy terms. Requests to access the datasets should be directed to VZ, veit.zebralla@medizin.uni-leipzig.de.

## Ethics Statement

The studies involving human participants were reviewed and approved by the Ethical Committee at the Medical Faculty, University of Leipzig (vote no. 201-10- 12072010 and no. 202-10-12072010).

## Author Contributions

VZ, AB, AD and SW contributed conception and design of the study; VZ, KB and KH organized the database; JM, SOJ and TN developed new visualization of the database; VZ, JM, TW and KB performed the statistical analysis; VZ wrote the first draft of the manuscript; SW, JM, TW and GW wrote sections of the manuscript. All authors contributed to the article and approved the submitted version.

## Conflict of Interest

TN is a shareholder of the software company IMPULS Technologiemanagement UG that provided the PROM software “OncoFunction”.

The remaining authors declare that the research was conducted in the absence of any commercial or financial relationships that could be construed as a potential conflict of interest.
